# HOXA-AS2 enhances GBM cell malignancy by suppressing miR-2116-3p thereby upregulating SERPINA3

**DOI:** 10.1186/s12885-022-09462-y

**Published:** 2022-04-06

**Authors:** Jianrui Sun, Lin Wang

**Affiliations:** 1 Department of Neurosurgery, The First Affiliated Hospital of Zhengzhou University, No. 1, Jianshe East Road, Zhengzhou, 450052 Henan China; 2 Information Department, The First Affiliated Hospital of Zhengzhou University, Zhengzhou, 450052 Henan China

**Keywords:** HOXA-AS2, miR-2116-3p, SERPINA3, Glioblastoma

## Abstract

**Background:**

Glioblastoma (GBM) is malignant, demanding more attention to the improvement of the diagnosis and therapy. LncRNAs have been implicated in the malignancy of GBM cells.

**Methods:**

HOXA-AS2, miR-2116-3p and SERPINA3 expression levels in GBM tissues and cell lines were detected by qRT-PCR. Western blotting was performed to detect the protein levels of Bax and Bcl-2. Dual-luciferase reporter assay was for detection of relationship among these factors, together with RIP and RNA pull-down. CCK-8, EdU, wound healing and transwell assays were for detection of the role of HOXA-AS2, miR-2116-3p and SERPINA3 in cell viability, proliferation, migration and invasion in GBM, respectively.

**Results:**

HOXA-AS2 and SERPINA3 showed higher level in GBM tissues and cell lines. Low level of HOXA-AS2 attenuated GBM cell growth in vitro. Moreover, the anti-tumor impact of silenced HOXA-AS2 was restored by miR-2116-3p inhibitor, but its tumor-promotional effect could be reversed by silenced SERPINA3.

**Conclusion:**

HOXA-AS2 enhanced GBM cell malignancy through sponging miR-2116-3p and releasing SERPINA3, which might shed light on the diagnosis and therapy for GBM in the future.

**Supplementary Information:**

The online version contains supplementary material available at 10.1186/s12885-022-09462-y.

## Introduction

Glioblastoma is an aggressive tumor that can trace its origin back to the brain or spinal cord, threatening a number of patients’ health and life [1, 2]. Despite constant efforts to devise new therapy strategies, therapy-resistant occurs and patients inescapably succumb to this thorny disease [3, 4]. Remarkably, demonstrations concerning non-coding RNA involved in the modulation of GMB progression are emerging, bringing good news to the improvement of treatment for GMB in recent years [5–8]. Therefore, attempt to expand our current knowledge on non-coding-RNA-regulatory action in GMB is without doubt of great significance. Here, we tried to address this problem by investigating whether and how non-coding RNA change the growth and activity of GMB cells.

Long non-coding RNAs (lncRNAs) is regarded as the molecules longer than 200 nt that have no potential to encode proteins [9, 10]. In last few years, this kind of molecule has attracted a widespread attention due to their potential role in modulation of carcinomatosis [11–13]. HOXA-AS2 has been linked to human carcinomatosis, including GBM. Early study reported by Guo et al. in 2018 depicted that reduced HOXA-AS2 restrained malignant glioma behaviors [14]. Subsequent study revealed a GBM-enhancing effect of HOXA-AS2 in low-grade recurrence in 2020 [15]. Although HOXA-AS2 has been depicted in the literature to deteriorate GBM, its regulatory mechanism is yet to be fully investigated.

It is well demonstrated that the small non-coding RNAs, miRNAs actively take part in post-transcriptional restriction or RNA degradation [16]. Over the past decades, available findings as well as emerging data depict that miRNAs possess active biological functions associated with the regulation of a variety of carcinomatosis [17–19]. Unlike many cancer-related miRNAs that have been extensively studied in various carcinoma types [20–22], the cancer-related role for miR-2116-3p is reported in limited published data so far. There is one published study that revealed the potential tumor-inhibiting action of miR-2116-3p in breast cancer [23]. But up to date, there is no research that explains the expression as well as the function of miR-2116-3p in the progression of GBM. Therefore, it is in need of the exploration of whether and how this miRNA acts in the progression of GBM.

Serpin peptidase inhibitor clade A member 3 (SERPINA3) is a protein coding gene [24]. Which encodes a plasma protease inhibitor belonging to the serine protease inhibitor class [24]. Mutations of the sequence of this protein have been implicated in Alzheimer’s disease [25]. What is noteworthy is that SERPINA3 has also been studied in a variety of carcinomas [26–29]. It is noteworthy that identification of biomarker candidates in plasma of GBM patients found SERPINA3 could serve as an essential serum biomarker for GBM diagnosis [30]. However, the mechanism of SERPINA3 affecting the development of GBM has not yet been clearly known.

We focused chiefly on the cross-talk and actions of the HOXA-AS2, miR-2116-3p and SERPINA3 in GBM cells. By this effort, it may be possible that the outcome of this research might provide new insights into understanding the pathological processes of GBM.

## Materials and methods

### Tissue sampling

GBM tumor tissues and adjacent normal tissues from 18 GBM patients were collected during operation from our hospital. Informed consent had been acquired before the operation. The performance was in line with the standards set by the Ethics Committee of this hospital.

### Cell culture and cell transfection

GBM cell lines (LN229, U251, A172, SHG44) and Normal Human Astrocytes (NHA) were purchased from American Type Culture Collection (ATCC, USA). The mentioned cells were maintained in DMEM (Solarbio, China) with 10% (v/v) FBS (Invitrogen, USA) and 1% penicillin-streptomycin solution (Sigma, USA) at 37 °C with 5% CO2. As for cell transfection, Lipofectamine 2000 (Invitrogen, USA) was applied here. For oligonucleotides, miR-2116-3p mimic, miR-2116-3p inhibitor, small interfering RNA against HOXA-AS2 (si-HOXA-AS2) or SERPINA3 (si-SERPINA3) and negative control were constructed by GenePharma (Shanghai, China). By the time the cells reached a density of around 70%, 50 nM aforementioned oligonucleotides were transected into U251 and A172 cells.

### qRT-PCR

Trizol reagent (Invitrogen, USA) was used for extraction for total RNA from tissues and cells. MonAmpTM SYBR® Green qPCR Mix (Monad, China) was used for detection of expression levels of HOXA-AS2, SERPINA3, and GAPDH. TaqMan MicroRNA Reverse Transcription kit and Taqman Universal Master Mix II (Applied Biosystems, USA) were used to detect miR-2116-3p and U6 expression. Relative expression values were normalized and calculated to represent fold change in gene expression using relative quantification as 2 − △△CT. The sequence information of primers was presented in Table 1.

**Table 1:** The sequences of the primers in this study

Primer	Sequences
**HOXA-AS2**	Forward: 5′-CCCGTAGGAAGAACCGATGA-3′
	Reverse: 5′-TTTAGGCCTTCGCAGACAGC-3′
**SERPINA3**	Forward: 5′-GACTCGCAGACAATGATGGTC-3′
	Reverse: 5′-GCAAACTCATCATGGGCACC-3′
**miR-2116-3p**	Forward: 5′-AATCCTATGCCAAGAACTCCC-3′
	Reverse: 5′-CTCTACAGCTATATTGCCAGCCA-3′
**GAPDH**	Forward: 5′-GAAGGTGAAAGGTCGGAGTC-3’
	Reverse: 5′-GAAGATGGTGATGGGATTTC-3’
**U6**	Forward: 5′-CTCGCTTCGGCAGCACATA-3’
	Reverse: 5′-AACGATTCACGAATTTGCGT-3’

### Subcellular fractionation

It was performed via mirVana™ PARIS™ Kit (Thermo Fisher Scientific, USA). The extraction of RNAs in U251 and A172 was conducted by Monarch® Total RNA Miniprep Kit (NEB, USA). Finally, qRT-PCR was for the determination for the enrichment of HOXA-AS2 in cytoplasmic and nuclear fractions.

### RNA fluorescence in situ hybridization (FISH)

HOXA-AS2 localization in U 251 and U172 cells was further verified using a RiboTM lncRNA FISH Probe Mix Kit (RiboBio, China) per its protocal. In short, 2× 10^5^ cells were plated on 24-well microplates precoated with coverslips. When grown into 85% confluence, cells were fixed with 4% paraformaldehyde for 10 min and then exposed to prehybridization solution at 37 °C. 30 min later, the prepared cells were detected with the HOXA-AS2 probe (RiboBio, China) at 37 °C. 24 h later, DAPI (Servicebio, China) counterstaining was performed to discriminate the nucleus from the cytoplasm. The staining samples were visualized under an LSM 5 Pascal Laser Scanning Microscope (Zeiss).

### CCK-8 assay

Cell Counting Kit-8 (GlpBio, USA) was utilized in this performance. U251 and A172 were seeded into 96-well plates (1 × 10^4^ cells/well) for the transfection. When the transfection time reached every 24 h from 0 h to 96 h, CCK-8 solution (10 μL) would be pipetted to each well for a 2-h incubation avoiding light. Subsequently, the OD (450 nm) was monitored in a microplate reader (Bio-Rad, USA).

### EdU assay

EdU assays were performed in proliferating U251 and A172 seeded in 24-well plates (5 × 105 cells/well). Briefly, 24 h after transfection, cells were incubated with 10 μM EdU (EdU Staining Proliferation Kit, Abcam) for another 24 h. The proliferating cells were visualized under a Leica DMi8 fluorescent microscope using the images of randomly selected fields obtained from the fluorescence microscope. The proliferation rate was determined by the numbers of EdU-stained cells (red) normalized to the numbers of Hoechst 33342-stained cells (blue).

### Wound healing assay

U251 and A172 were cultured in 6-well plates (2.5 × 106 cells/well). A scratch was generated by a 200-μL pipette tip at the point that the cells were grew to a single layer. The non-attached cells were washed away with washing of PBS buffer. The migrated cells moved to the wound 24 h after the scratching was captured applying an inverted microscope (Olympus, Japan).

### Transwell assay for invasion detection

The invasion ability of U251 or A172 cells was assessed using Matrigel-Coated Transwell Chambers (BD Bioscience). Cells were seeded into the upper chamber. After overnight incubation, cells in lower chamber were fixed with 4% paraformaldehyde, followed by staining with 0.1% crystal violet. They were counted under a microscope (Olympus, Japan).

### Dual-luciferase reporter assay

Wild-type or mutant HOXA-AS2 or SERPINA3 dual-luciferase reporter (100 ng) and miR-2116-3p mimic or NC duplexes (50 nM) were co-transfected into U251 or A172 cells in a 96-well plate. Firefly and Renilla luciferase activities were measured after 48 h transfection using a Dual-GLO Luciferase Assay System (Promega, USA).

### RIP assay

Imprint RNA immunoprecipitation kit (Sigma, USA) was applied to do this detection. The cells were treated with RIP lysis buffer provided by the kit at the point of 48-h post-transfection. Before that, magnetic beads were precoated with antibody of Ago2 or IgG, which was then added to the collected cell lysis for incubation of 4 h. Following being cleaned, the immunoprecipitated RNA was extracted by TRIzol reagent. The relative enrichment of SERPINA3 was measured by qRT-PCR. The sequence of miR-2116-3p is CCUCCCAUGCCAAGAACUCCC.

### RNA pull-down

An RNA pull-down assay was carried out in accordance with Pierce RNA 3′ End Desthiobiotinylation Kit (Thermo Fisher Scientific, USA). In brief, biotinylated probe was incubated with magnetic beads (Life Technologies) to generate probe-coated beads, which was later blended with the cell lysates overnight. Being washing by wash buffer, the RNA was extracted from the complexes by RNeasy Mini Kit (QIAGEN, Germany). qRT-PCR assay that was for the measurement of SERPINA3 level. The sequence of miR-2116-3p mimic is 5′-CCUCCCAUGCCAAGAACUCCC-3′.

### Western blotting

Extraction of total protein was performed using PIPA Buffer (Beyotime, China). Separation of the proteins was carried out via 10% SDS gel electrophoresis. Then, the proteins were transferred to the PVDF membranes (BioRad, USA). Antibodies SERPINA3 (Cat#: ab184567, Abcam, UK), anti-Bax (1:1000, Cat#: ab53154, Abcam, UK), anti-Bcl-2 (1:1000, Cat#: ab32124, Abcam, UK), anti-caspase-3 (1:1000, Cat#: ab32351, Abcam, UK), anti-cleaved caspase-3 (1:1000, Cat#: ab2302, Abcam, UK) and GAPDH (Cat#: ab8245, Abcam, UK) were used to incubate the membranes with dilution 1:1000 at 4 °C overnight. The other day, the samples were treated with secondary antibodies. The signal of protein was detected with the chemiluminescence (Amersham Life Science, UK).

### Statistical analysis

All performances were at least triple. Data were presented as mean ± SD. All statistical calculations were performed in GraphPad Prism version 8. Two-tailed Student’s t-test was for two groups and one-way ANOVA was for multiple comparisons. *P < 0.05, **P < 0.001 was considered as statistically significant difference.

## Result

### HOXA-AS2 might be a key lncRNA of GBM

**Fig. 1:**
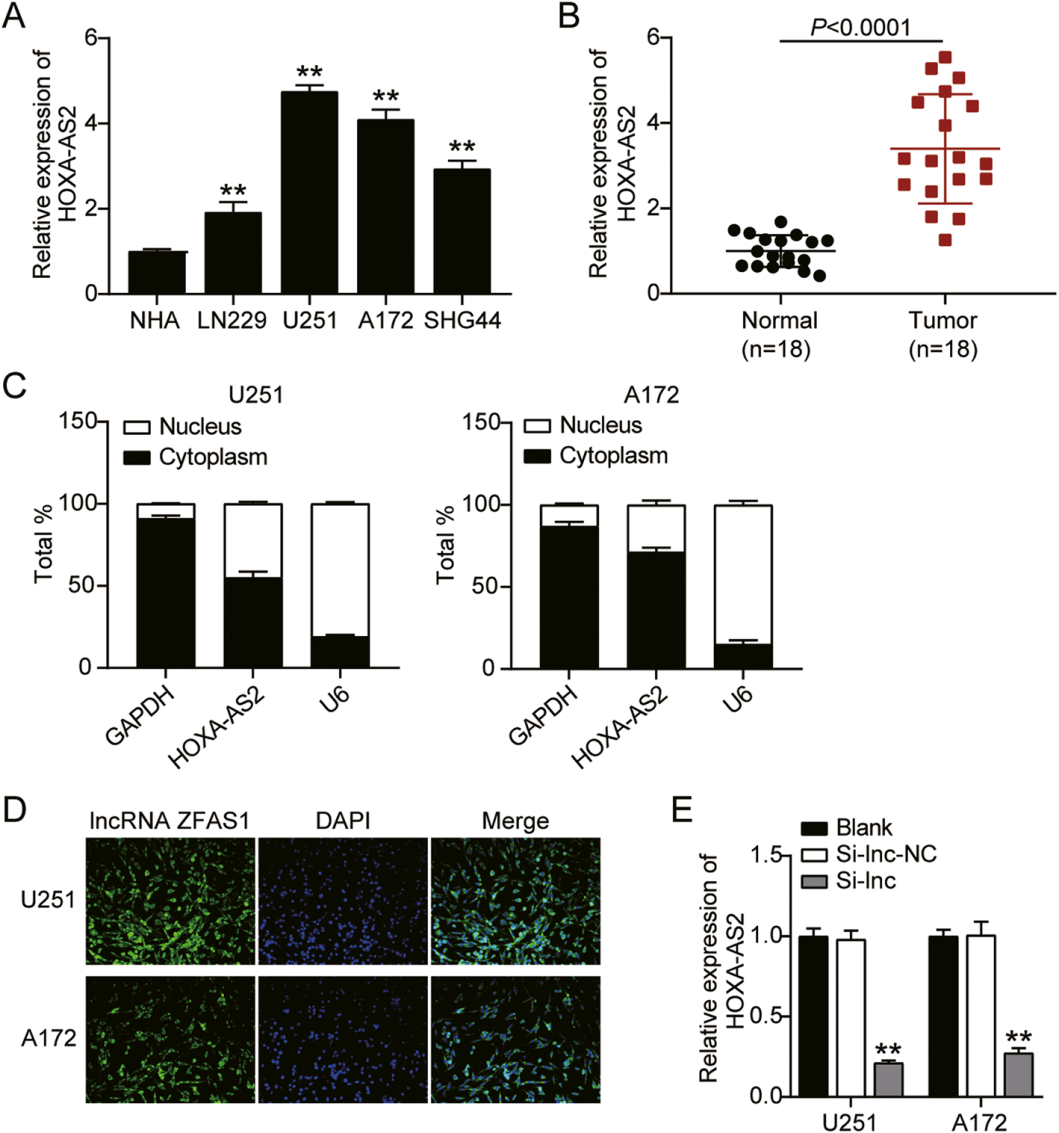
HOXA-AS2 might be a key lncRNA of GBM. **A** HOXA-AS2 expression in GBM cell lines (LN229, U251, A172, and SHG44) and Normal human Astrocytes (NHA) by qRT-PCR. **P < 0.001 compared with NHA. **B** HOXA-AS2 expression in GBM tissues and corresponding normal tissues by qRT-PCR. *P* < 0.0001 compared with normal tissues. **C** The intracellular distribution of HOXA-AS2 in U251 and A172 cells by subcellular fractionation. **D** Representative images of FISH showing the subcellular localization of HOXA-AS2 in U251 and A172 cells **E** The transfection efficiency of si-HOXA-AS2 in U251 and A172 cells by qRT-PCR. **P < 0.001 compared with blank group. Data were present as mean ± SD

HOXA-AS2 has been reported to enhance GBM malignancy. As shown in Fig. 1A, compared with NHA cells, HOXA-AS2 expression in the GBM cell lines (LN229, U251, A172, and SHG44) was significantly increased, with U251 and A172 exhibiting the most significant increase (more than 4-fold relative to NHA). Therefore, U251 and A172 were chosen for the subsequent analysis. Additionally, as shown in Fig. 1B, contrasted with adjacent normal tissues, HOXA-AS2 expression in tumor samples exhibited a significant elevation (almost 4-fold). Moreover, the distribution of HOXA-AS2 in GBM cell lines was detected and the result showed that HOXA-AS2 mainly distributed in cell cytoplasm (Fig. 1C and Fig. 1D). HOXA-AS2 was successfully knocked down (by about 70%) in U251 and A172 (Fig. 1E). Taken together, HOXA-AS2 might be a key lncRNA in GBM.

### HOXA-AS2 promoted GBM cell tumor growth and inhibited cell apoptosis

**Fig. 2:**
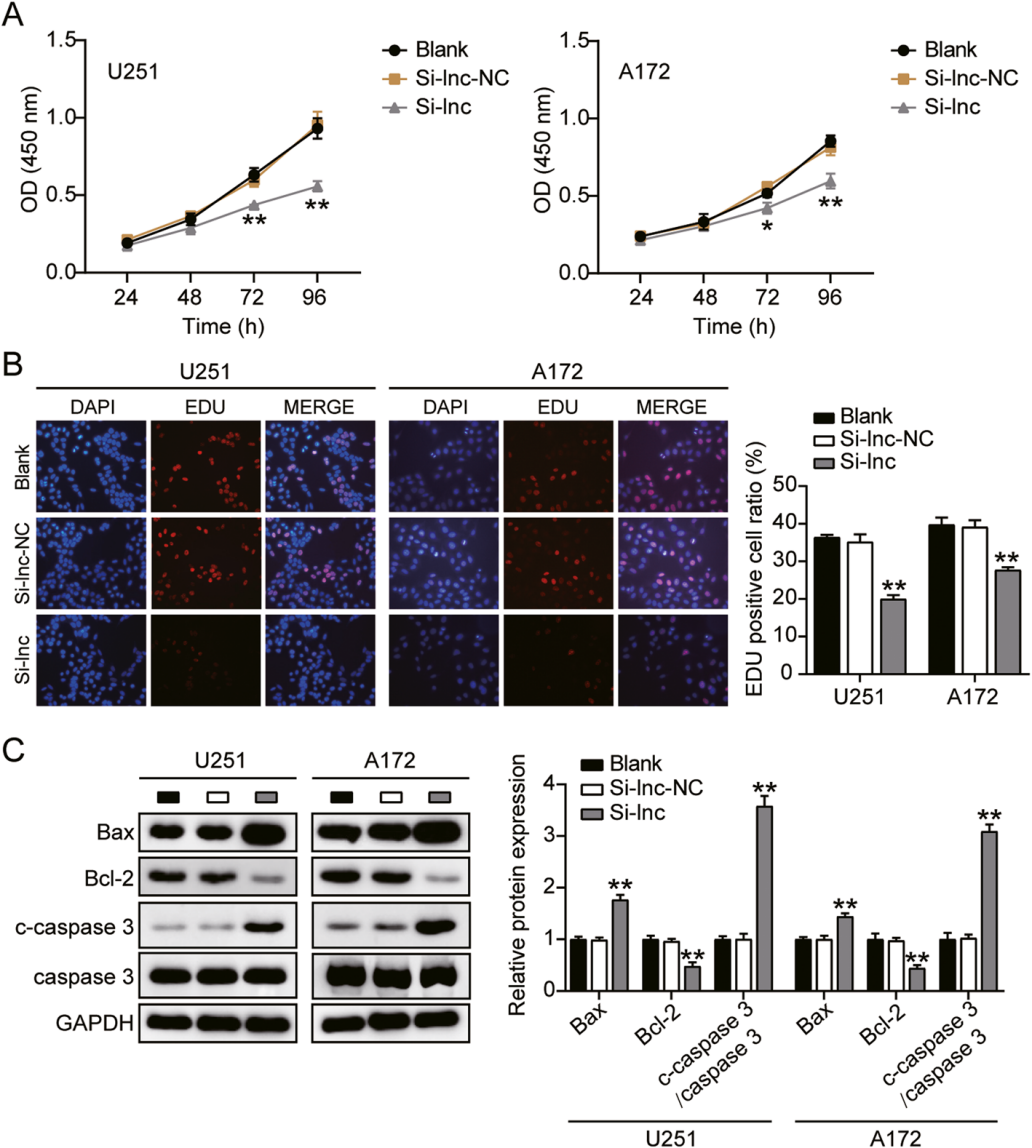
HOXA-AS2 promoted GBM cell proliferation and inhibited cell apoptosis. **A** Cell viability was detected by CCK-8 assay. **B** Cell proliferation was detected by EdU assay. **C** The protein levels of Bax, Bcl-2, cleaved-caspase-3, caspase-3 in GBM cells were detected by western blotting. (A-C) Cells were transfected with si-HOXA-AS2. *P < 0.05, **P < 0.001 compared with blank group. Data were present as mean ± SD

Given that HOXA-AS2 might be involved in the tumor growth of GBM, we carried out the following confirmations. Silenced HOXA-AS2 significantly reduced cell viability of U251 and A172 (Fig. 2A). HOXA-AS2 knockdown repressed cell proliferation of U251 by around 40% and A172 by 30% compared to the control group (Fig. 2B). Compared to the blank group, silenced HOXA-AS2 elevated the protein expression of apoptosis-promoting gene Bax and cleaved-caspase-3 by almost 70% in U251 and about 50% in A172 cells. But silenced HOXA-AS2 reduced the protein expression of anti-apoptosis gene Bcl-2 by about 50% in U251 and A172 (Fig. 2C). These results suggested that silenced HOXA-AS2 induced cell apoptosis of GBM cells. These results suggested HOXA-AS2 functionally facilitated GBM cell tumor growth.

### HOXA-AS2 promoted GBM cell migration and invasion

**Fig. 3:**
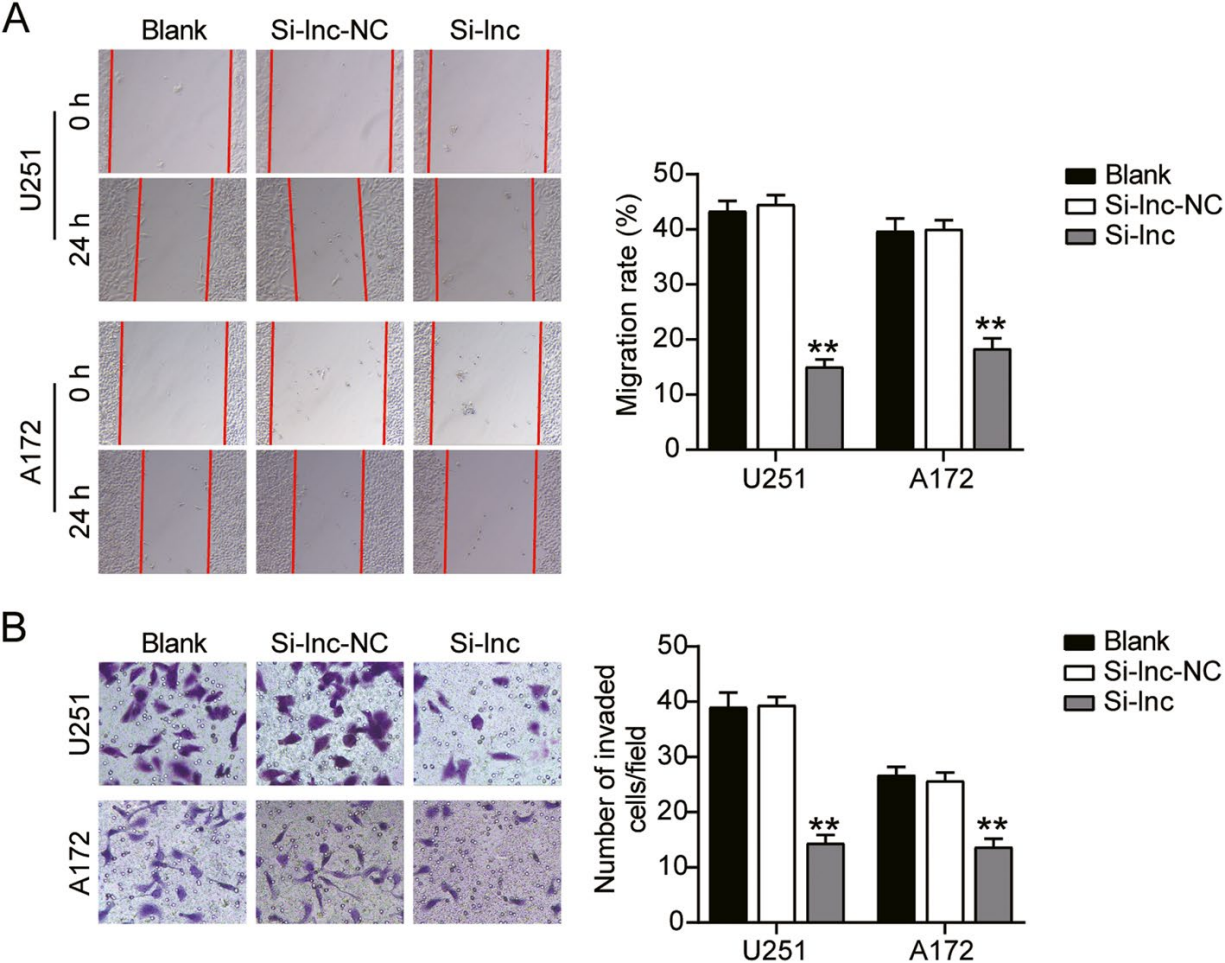
HOXA-AS2 promoted GBM cell migration and invasion. **A** Cell migration ability was detected by wound healing assay. **B** Cell invasion was detected by transwell assay. (A-B) Cells were transfected with si-HOXA-AS2. *P < 0.05, **P < 0.001 compared with blank group. Data were present as mean ± SD

Cell migration and invasion were two important processes in cancer development. Thus we continued to monitor how HOXA-AS2 acted on these two processes. As a result, compared to the control group, knockdown of HOXA-AS2 induced 60% inhibition of cell migration in U251 and 50% inhibition of cell migration in A172 (Fig. 3A). Knockdown of HOXA-AS2 induced 60% inhibition of cell invasion in U251 and 30% inhibition of cell invasion in A172 (Fig. 3B). These results suggested HOXA-AS2 functionally facilitated GBM cell migration and invasion.

### Established the downstream of HOXA-AS2

Considering its enrichment in the cytoplasm of HOXA-AS2 in GBM cells, we planned to investigate the downstream of HOXA-AS2 by miRNA/mRNA axis. SERPINA3 was the most significantly upregulated gene in GBM (Supplementary Fig. 1A) according to GEPIA GBM data (http://gepia2.cancer-pku.cn/#degenes). After reviewing the literatures, SERPINA3 was reported to be overexpressed in GBM tissues and associated with the poor prognosis outcome of GBM patients [30–32], to stimulate stem-like properties of GBM cells [31, 33], and the knockdown of SERPINA3 significantly caused the suppressed GBM cell invasion [33]. These studies strongly suggest that SERPINA3 could play a significant role in GBM progression. Then, we used starbase algorithm and TargetScan Human 7.2 software to screen the miRNA biding to HOXA-AS2 and SERPINA3, respectively. The result showed that miR-2116-3p was an only one miRNA to link HOXA-AS2 and SERPINA3 mRNA (Supplementary Fig. 1B). Therefore, we explore if the action of HOXA-AS2 required miR-2116-3p/ SERPINA3 axis.

### HOXA-AS2 sponged miR-2116-3p in GBM

**Fig. 4:**
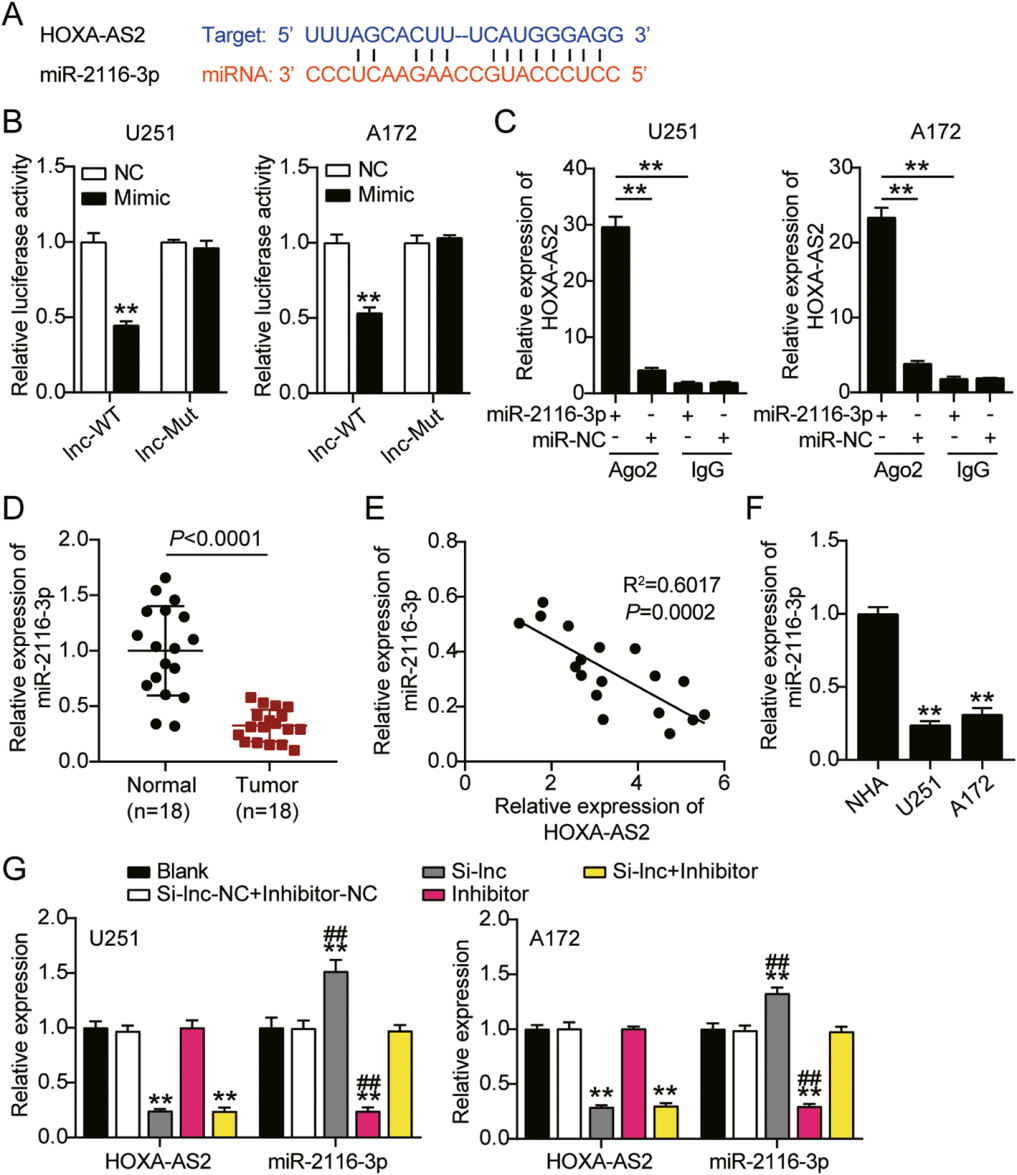
HOXA-AS2 sponged miR-2116-3p in GBM. **A** The potential matching site between HOXA-AS2 and miR-2116-3p was predicted. **B** Dual-luciferase reporter assay was performed to verify the relationship between HOXA-AS2 and miR-2116-3p. **P < 0.001 compared with miR-2116-3p mimic NC. **C** HOXA-AS2 could be obviously pulled down by miR-2116-3p mimic showed by RIP analysis. **P < 0.001 compared with anti-IgG. **D** Lower expression of miR-2116-3p in GBM tissues. **P < 0.001 compared with normal tissues. **E** Negative correlation between HOXA-AS2 and miR-2116-3p expression. **F** Lower expression of miR-2116-3p in U251 and A172 cells. **P < 0.001 compared with NHA. **G** The transfection efficiency of miR-2116-3p inhibitor in U251 and A172 cells by qRT-PCR. *P < 0.05, **P < 0.001 compared with blank group. #*P* < 0.05, ##*P* < 0.001 compared with Si-lnc + inhibitor group. Data were present as mean ± SD

Our prediction through the online prediction tool Starbase v2.0 displayed HOXA-AS2 sponged miR-2116-3p in GBM (Fig. 4A). To validate the predicted results, luciferase reporter assay was performed using HOXA-AS2 WT or HOXA-AS2 MUT luciferase reporter plasmid. The luciferase activity of HOXA-AS2 WT was reduced by miR-2116-3p mimic, but there was no significant change in the luciferase activity of mutant group (Fig. 4B). It was confirmed that HOXA-AS2 could be dramatically pulled down by anti-Ago2 in U251 and A172 (Fig. 4C). Moreover, miR-2116-3p expression in GBM tissues was 2-fold lower in normal tissues, having an opposite level of HOXA-AS2 in GBM tissues (Fig. 4D). Furthermore, a significant negative correlation (R2 = 0.535) between HOXA-AS2 and miR-2116-3p was observed in GBM (Fig. 4E). As shown in Fig. 4F, miR-2116-3p showed a significant reduced level in GBM cell lines (70% in U251 and 60% in A172). From Fig. 4G, we could see that miR-2116-3p was successfully knocked down by miR-2116-3p inhibitor, indicating it could be used in the following experiments. Collectively, HOXA-AS2 sponged miR-2116-3p in GBM.

### The effects of HOXA-AS2 were achieved by regulation on miR-2116-3p

**Fig. 5:**
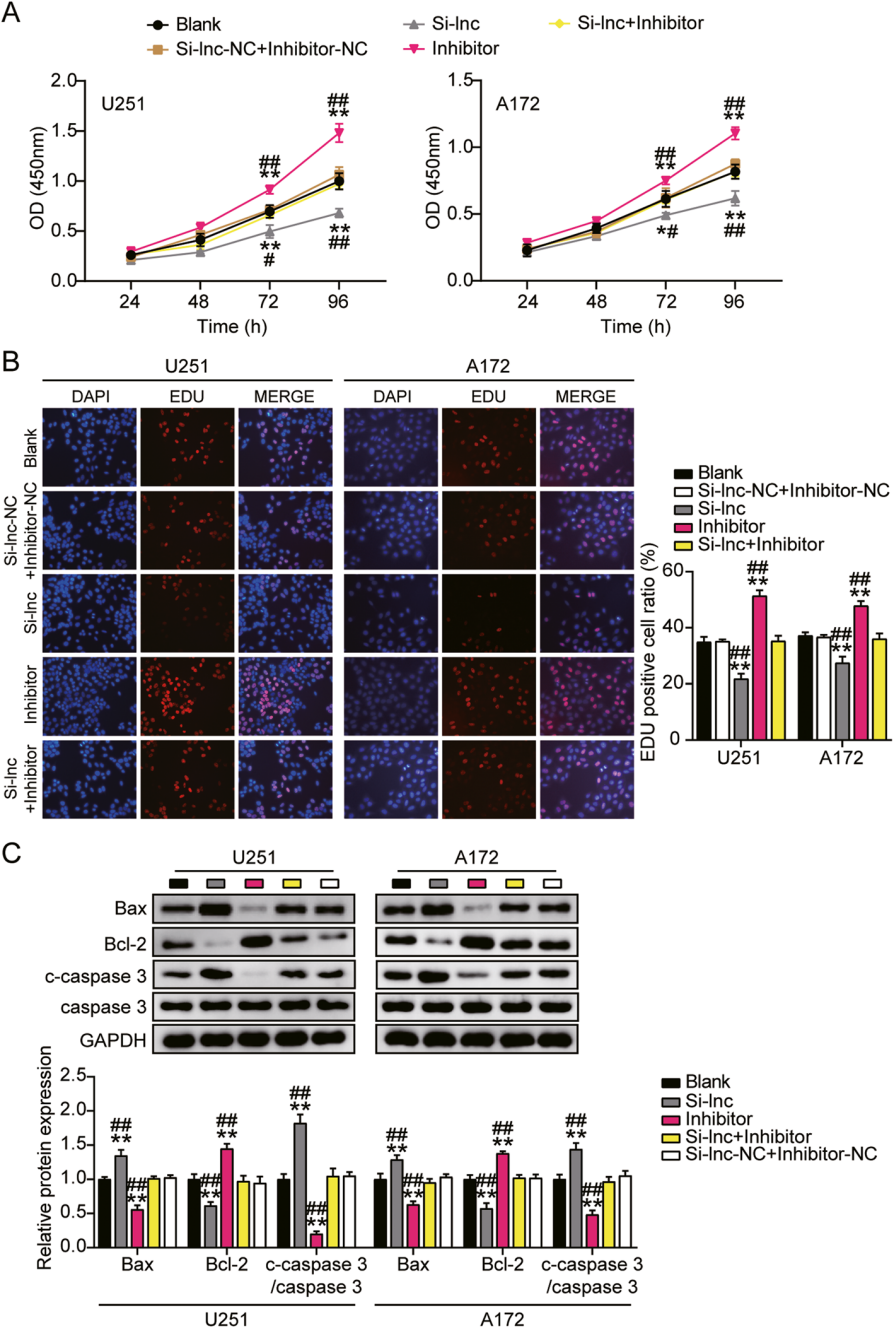
The effects of HOXA-AS2 were achieved by regulation on miR-2116-3p. **A** CCK-8 assay was performed to detect cell viability. **B** EdU assay was used to detect cell proliferation. **C** The protein levels of Bax, Bcl-2, cleaved-caspase-3, caspase-3 in GBM cells were detected by western blotting. (A-C) U251 and A172 cells were transfected with si-HOXA-AS2, miR-2116-3p inhibitor, si-HOXA-AS2 plus miR-2116-3p inhibitor or negative control. *P < 0.05, **P < 0.001 compared with blank group. #*P* < 0.05, ##*P* < 0.001 compared with Si-lnc + inhibitor group. Data were present as mean ± SD

The miR-2116-3p inhibitor and si-HOXA-AS2 were co-transfected into U251 and A172. Contrasted with the blank group, si-HOXA-AS2 exhibited great inhibition on cell viability, and the existence of miR-2116-3p inhibitor partially abolished the actions of si-HOXA-AS2 in cell viability (Fig. 5A). Similarly, relative to the blank group, si-HOXA-AS2 exerted a dramatical suppression on cell proliferative ability, and this inhibition could be partially abolished by miR-30a-5p inhibitor (Fig. 5B). On the other hand, cell apoptosis was induced by si-HOXA-AS2, while this induction could be restored by miR-2116-3p inhibitor (Fig. 5C). Specifically, silenced miR-2116-3p decreased the protein expression of apoptosis-promoting gene Bax and cleaved-caspase-3 by almost 50% in U251 and about 40% in A172 cells. But silenced miR-2116-3p induced the protein expression of anti-apoptosis gene Bcl-2 by about 50% in U251 and A172 (Fig. 5C). These results indicated that HOXA-AS2 improved cell viability, proliferation of GBM cell lines and restrained cell apoptosis by modulating miR-2116-3p.

### miR-2116-3p inhibited GBM cell migration and invasion

**Fig. 6:**
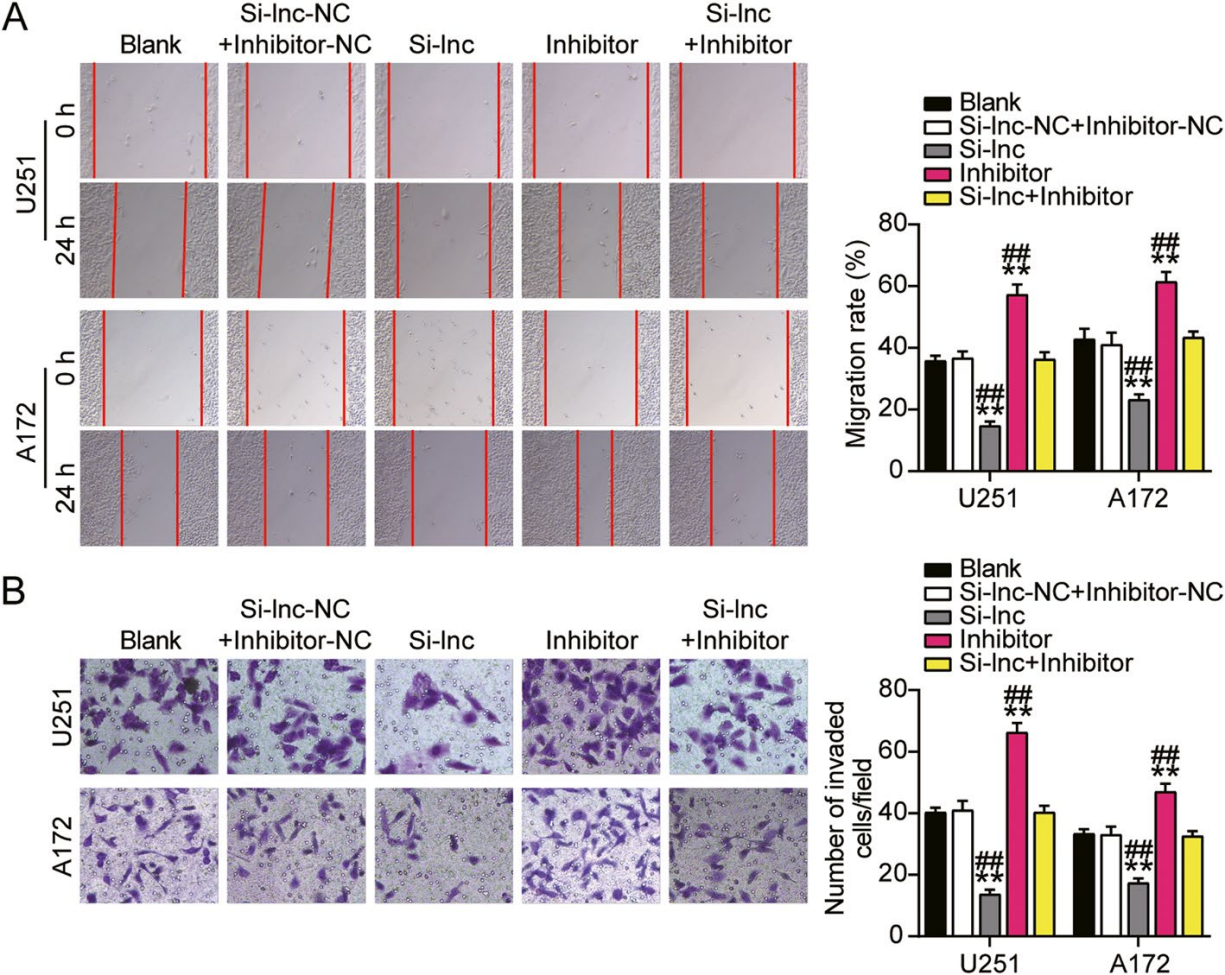
miR-2116-3p inhibited GBM cell migration and invasion. **A** Wound healing assay was used to detect cell migration. **B** Cell invasion was detected by transwell assay. (A-B) U251 and A172 cells were transfected with si-HOXA-AS2, miR-2116-3p inhibitor, si-HOXA-AS2 plus miR-2116-3p inhibitor or negative control. *P < 0.05, **P < 0.001 compared with blank group. #*P* < 0.05, ##*P* < 0.001 compared with Si-lnc + inhibitor group. Data were present as mean ± SD

Compared to the control group, knockdown of HOXA-AS2 induced 50% inhibition of cell migration in U251 and 30% in A172, while the inhibition caused by si-HOXA-AS2 could be reversed by miR-2116-3p inhibitor (Fig. 6A). Besides, 60% inhibition of cell invasion in U251 and 50% in A172 resulted in si-HOXA-AS2 could be also reversed by miR-2116-3p inhibitor (Fig. 6B). These results suggested HOXA-AS2 functionally regulated GBM cell malignancy through interaction with miR-2116-3p.

### SERPINA3 was a target of miR-2116-3p

**Fig. 7:**
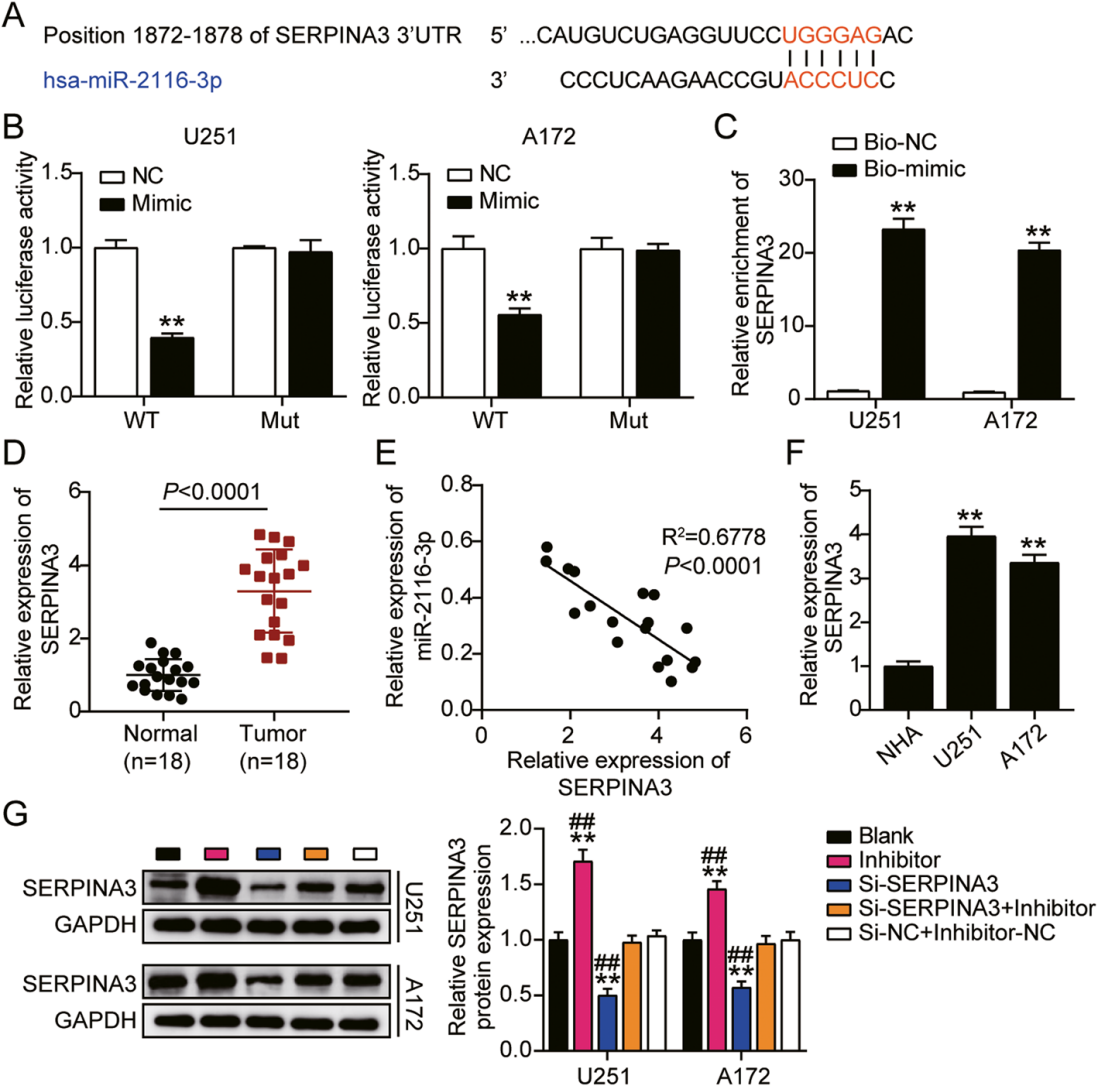
SERPINA3 was a target of miR-2116-3p. **A** The potential binding sites between miR-2116-3p and SERPINA3. **B** Dual-luciferase reporter assay was performed to verify the relationship between miR-2116-3p and SERPINA3. **P < 0.001 compared with miR-2116-3p mimic NC. **C** SERPINA3 could be obviously pulled down by miR-2116-3p mimic showed by RNA pull-down analysis. **P < 0.001 compared with bio-mimic-NC. **D** Upregulation of SERPINA3 in GBM tissues. **P < 0.001 compared with normal tissues. (E) Negative correlation between miR-2116-3p and SERPINA3 expression. **F** Upregulation of SERPINA3 in U251 and A172 cells. **P < 0.001 compared with NHA. **G** The transfection efficiency of si-SERPINA3 in U251 and A172 cells by western blotting. *P < 0.05, **P < 0.001 compared with blank group. #*P* < 0.05, ##*P* < 0.001 compared with Si-SERPINA3 + inhibitor group. Data were present as mean ± SD

SERPINA3 was predicted to be a target of miR-2116-3p (Fig. 7A). The luciferase activity of SERPINA3 WT plus miR-2116-3p mimic group was obviously restrained, but no significant change was observed in SERPINA3 MUT plus miR-2116-3p mimic group, proving the direct target relationship between SERPINA3 and miR-2116-3p (Fig. 7B). Also, it is determined that SERPINA3 was significantly pulled down by bio-miR-2116-3p in U251 and A172 (Fig. 7C). SERPINA3 expression in GBM tissues was 3.5-fold higher in normal tissues (Fig. 7D), showing a negative correlation with miR-2116-3p expression (Fig. 7E). As shown in Fig. 7F, SERPINA3 showed a significant increased level in GBM cell lines (U251 and A172). Furthermore, as shown in Fig. 7G, miR-2116-3p inhibitor significantly increased SERPINA3 protein expression level about 65% in U251 and 50% in A172, and SERPINA3 knockdown significantly decreased the expression level of SERPINA3 protein by about 50%. These results indicated that miR-2116-3p exerted its function through inhibition on SERPINA3 in GBM.

### miR-2116-3p exerted its function through mediation on SERPINA3 in GBM

**Fig. 8:**
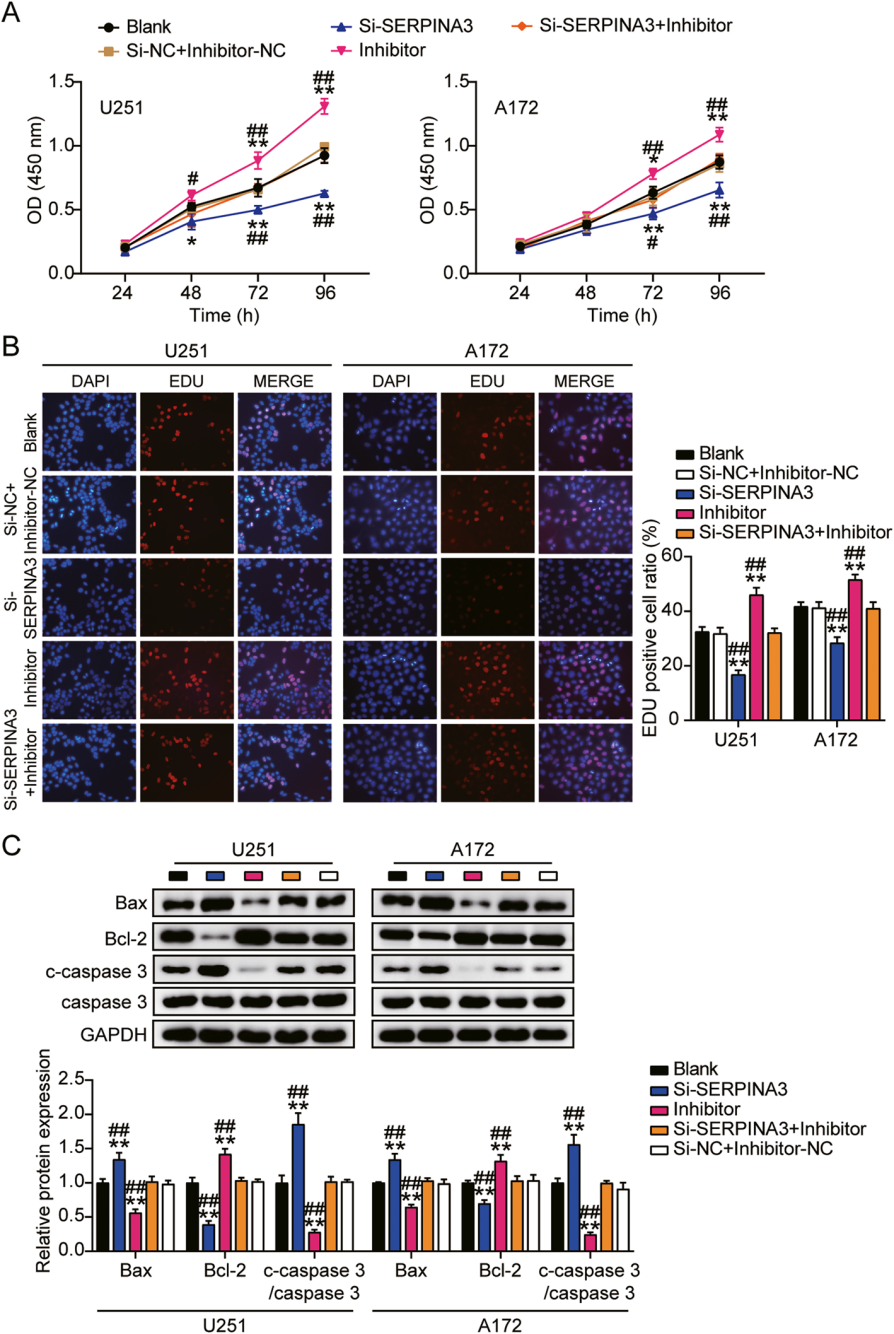
miR-2116-3p exerted its function through mediation on SERPINA3 in GBM. **A** CCK-8 assay was performed to detect cell viability. **B** EdU assay was used to detect cell proliferation. **C** The protein levels of Bax, Bcl-2, cleaved-caspase-3, caspase-3 in GBM cells were detected by western blotting. (A-C) U251 and A172 cells were transfected with si-SERPINA3, miR-2116-3p inhibitor, si-SERPINA3 plus miR-2116-3p inhibitor or negative control. *P < 0.05, **P < 0.001 compared with blank group. #*P* < 0.05, ##*P* < 0.001 compared with Si-SERPINA3 + inhibitor group. Data were present as mean ± SD

Similar to the confirmation of the mechanism by which HOXA-AS2 regulated GBM cell tumor growth, The miR-2116-3p inhibitor and si-SERPINA3 were co-transfected into U251 and A172. Contrasted with the control group, miR-2116-3p inhibitor had a promotional effect on cell viability, while si-SERPINA3 could partially compromise the promotion of miR-2116-3p inhibitor on cell viability (Fig. 8A). Compared with the control group, miR-30a-5p inhibitor had an enhanced impact on cell proliferative ability, and this enhancement could be partially abolished by si-SERPINA3 (Fig. 8B). Cell apoptosis was reduced by miR-2116-3p inhibitor while this reduction could be restored by si-SERPINA3 (Fig. 8C). More specifically, si-SERPINA3 increased the protein expression of apoptosis-promoting gene Bax and cleaved-caspase-3 by about 40% in U251 and A172 cells. But si-SERPINA3 reduced the protein expression of anti-apoptosis gene Bcl-2 by about 60% in U251 and 30% in A172 (Fig. 8C). These results suggested miR-2116-3p exerted its function through mediation on SERPINA3 in GBM.

### SERPINA3 facilitated GBM cell migration and invasion

**Fig. 9:**
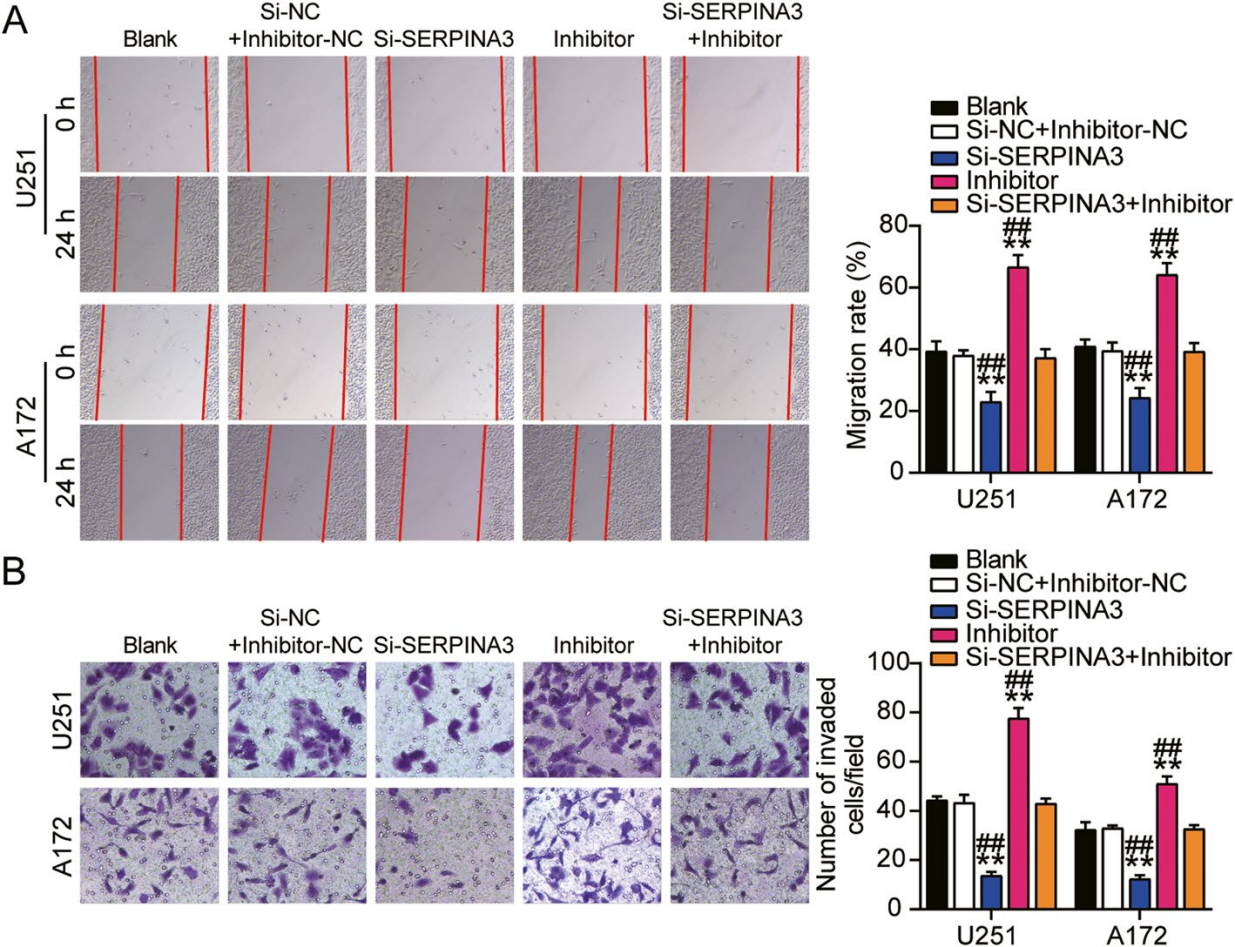
SERPINA3 facilitated GBM cell migration and invasion. **A** Wound healing assay was used to detect cell migration. **B** Cell invasion was detected by transwell assay. (A-B) U251 and A172 cells were transfected with si-SERPINA3, miR-2116-3p inhibitor, si-SERPINA3 plus miR-2116-3p inhibitor or negative control. *P < 0.05, **P < 0.001 compared with blank group. #*P* < 0.05, ##*P* < 0.001 compared with Si-SERPINA3 + inhibitor group. Data were present as mean ± SD

Compared to the control group, knockdown of miR-2116-3p led to 70% promotion of cell migration, while the promotion caused by miR-2116-3p inhibitor could be reversed by si-SERPINA3 (Fig. 9A). Besides, 70 and 50% induction of cell invasion caused by miR-2116-3p inhibitor could be also reversed by si-SERPINA3 (Fig. 9B). These results suggested miR-2116-3p exerted its function through mediation on SERPINA3 in GBM cell migration and invasion.

## Discussion

Herein, lncRNA HOXA-AS2 was identified to show a high enrichment in GBM, mediating miR-2116-3p/SERPINA3 in GBM cell malignancy. Knockdown of HOXA-AS2 could suppress cell viability as well as cell proliferative ability, further influencing apoptotic cell numbers, migration and invasiveness in GBM. On the other hand, miR-2116-3p knockdown in GBM cells revealed contrary tendency. Moreover, we proved that HOXA-AS2 could release the inhibition on SERPINA3 resulted from miR-2116-3p in the regulation of GBM cell malignancy. All in all, our results suggested that HOXA-AS2/miR-2116-3p/SERPINA3 could be used as novel markers of GBM and were potential therapeutic targets for GBM treatment.

HOXA-AS2 has been well established to be remarkably highly present in various tumor carcinogenesis including prostate cancer [34], non-small cell lung cancer [35] and type I endometrial carcinoma [36]. In the above-mentioned cancer progresses, HOXA-AS2 has been characterized to exert a potent tumor-stimulative function and have the potential to serve as a promising biomarker for predicting the risk of metastasis and mortality in cancer development. In our present study, we as well observed that remarkable enrichment of HOXA-AS2 was not only found in GBM tumor tissues but also in the GBM cell lines. Furthermore, by carrying out a series of in vitro verification, we also disclosed that HOXA-AS2 acted as a crucial regulator in GBM cells, facilitating GBM cell viability, proliferation, migration and invasion, while suppressing cell apoptosis.

Over the past decade, lncRNAs having the ability to sponge miRNA and then modulate the miRNA targets has been well discussed [37]. Here, we proved that miR-2116-3p contained binding sites for HOXA-AS2. What’ s more, the repressed cell viability in GBM cells by silenced HOXA-AS2 could be compromised by miR-2116-3p inhibitor. In addition, same effects were observed in the regulation of cell proliferation, cell migration and invasion. The attenuated function of silenced HOXA-AS2 could all be reversed by introducing the miR-2116-3p inhibitor in GBM cells. Furthermore, we measured the protein expression levels of Bax and Bcl-2 in the GBM cells after challenged to si-HOXA-AS2 and/or miR-2116-3p inhibitor. It is well demonstrated that Bax and Bcl-2 are two important genes that belong to the Bcl-2 protein family [38]. The protein encoded by Bax is an important apoptotic activator, and its overexpression is able to antagonize the cell-protective effect of Bcl-2 in the regulation of cell apoptosis [39]. The association and the ratio of Bax to Bcl-2 also determines survival or death of a cell following an apoptotic stimulus [40]. Our results demonstrated that si-HOXA-AS2 enhanced Bax protein expression level while reduced Bcl-2 protein expression in GBM cells. On the contrary, miR-2116-3p inhibitor repressed Bax protein expression level while induced Bcl-2 protein expression in GBM cells. These results depicted that si-HOXA-AS2 promoted cell apoptosis while miR-2116-3p inhibitor suppressed cell apoptosis in GBM cells.

To the best of our knowledge, our present research is the first to reveal the role of miR-2116-3p in GBM. Unlike the well-established role of HOXA-AS2 in many cancer development progressions, only few demonstrations were concerning the function of miR-2116-3p in cancer process. One published study revealed the potential tumor-inhibiting action of miR-2116-3p in breast cancer [23]. In the current study, we revealed the tumor-inhibiting role of miR-2116-3p in GBM cells, which is in line with the previous study. What’s more, we proved the action of miR-2116-3p in GBM cell malignancy could be regulated by HOXA-AS2. Besides, the downstream gene of miR-2116-3p (SERPINA3) was identified in GBM cells.

SERPINA3 has been previously found in the plasma of GBM patients [30], which indicated the potential promotional role of SERPINA3 in GBM development. However, its detailed regulatory roles in GBM development has not been well studied. Here, we knocked down the expression of SERPINA3 in GBM cells and observed its influence on GBM cell viability, proliferation, migration and invasion with knockdown of miR-2116-3p. Our findings revealed that si-SERPINA3 could partially compromise the promotion of miR-2116-3p inhibitor on cell viability and cell proliferation. Furthermore, si-SERPINA3 increased the protein expression of apoptosis-promoting gene Bax while reduced the protein expression of anti-apoptosis gene Bcl-2, which is opposite to that of miR-2116-3p. In addition, the promotion on cell migration and invasion caused by miR-2116-3p inhibitor could be reversed by si-SERPINA3. These results suggested the function of SERPINA3 in GBM cells was regulated by miR-2116-3p.

Sustaining proliferation, apoptosis, invasion and migration are characteristic features in the majority of malignant tumors [41]. Mounting evidence has focused on these cell phenotypes to mine the underlying mechanism. In this study, we did not investigate whether cells dying or primed to die resulted in migration and invasion, because we just observed the alternation in cell phenotypes. We will explore whether cells dying or primed to die resulted in migration and invasion in the future.

## Conclusion

In conclusion, HOXA-AS2 showed an elevated level in GBM cell malignancy. HOXA-AS2 could act as a molecular sponge of miR-2116-3p and significantly contributed to GBM cell proliferation, migration and invasion by activating the protein expression of SERPINA3. The current demonstration of the HOXA-AS2/miR-2116-3p/SERPINA3 axis could provide more effective clinical therapeutic strategy for GBM patients.

## Supplementary Information

**Additional file 1: Supplementary Figure 1.** Bioinformatics analysis identified SERPINA3 and miR-2116-3p. (A) The expression of SERPINA3 in GBM was analyzed by GEPIA. (B) miR-2116-3p was a common miRNA in starBase and TargetScan. starBase, a tool to predict miRNAs binding to HOXA-AS2. TargetScan, a tool to predict miRNAs binding to SERPINA3.

**Additional file 2.** Explaination.

**Additional file 3.** Western blotting.

## Data Availability

The datasets used and/or analyzed during the current study are available from the corresponding author on reasonable request.

## Abbreviations

GBM: Glioblastoma
lncRNAs: Long non-coding RNAs
SERPINA3: Serpin peptidase inhibitor clade A member 3
