# Correction: HOXA-AS2 enhances GBM cell malignancy by suppressing miR-2116-3p thereby upregulating SERPINA3

**DOI:** 10.1186/s12885-022-09579-0

**Published:** 2022-04-28

**Authors:** Jianrui Sun, Lin Wang

**Affiliations:** 1 Department of Neurosurgery, The First Affiliated Hospital of Zhengzhou University, No. 1, Jianshe East Road, Zhengzhou, 450052 Henan China; 2 Information Department, The First Affiliated Hospital of Zhengzhou University, Zhengzhou, 450052 Henan China

**Correction to: BMC Cancer 22, 366 (2022)**

**https://doi.org/10.1186/s12885-022-09462-y**

Following publication of the original article [1], the authors identified an error in the contributions section. The correct contributions section should state:

All contributions were made by JS and LW. The authors read and approved the final manuscript.
