# Supplementary material for: HOXA-AS2 enhances GBM cell malignancy by suppressing miR-2116-3p thereby upregulating SERPINA3
Source: BMC Cancer. 2022 Apr 6;22:366. doi: 10.1186/s12885-022-09462-y (PMC8985346; doi:10.1186/s12885-022-09462-y)
Supplement: Supplementary file 1 — Additional file 1: Supplementary Figure 1. Bioinformatics analysis identified SERPINA3 and miR-2116-3p. (A) The expression of SERPINA3 in GBM was analyzed by GEPIA. (B) miR-2116-3p was a common miRNA in starBase and TargetScan. starBase, a tool to predict miRNAs binding to HOXA-AS2. TargetScan, a tool to predict miRNAs binding to SERPINA3. [file 12885_2022_9462_MOESM1_ESM.docx]

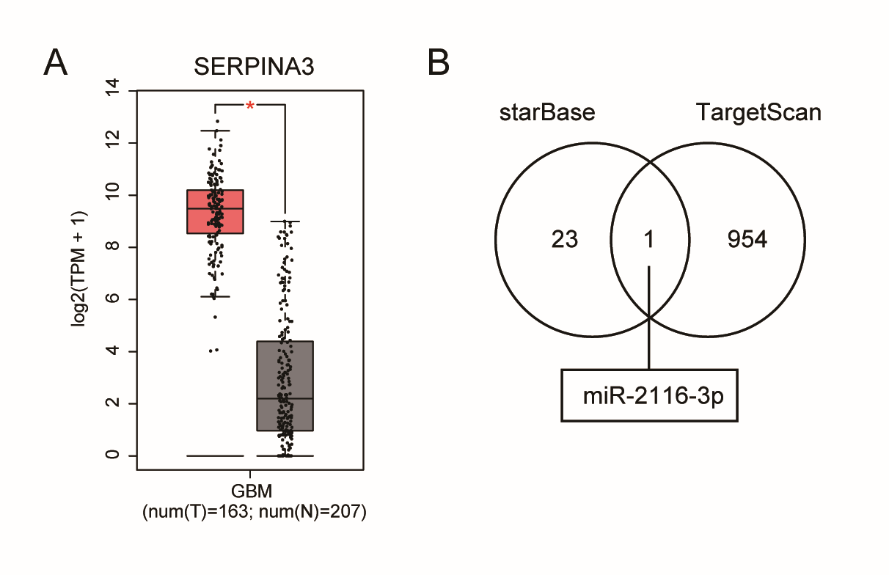


**Supplementary Figure 1. Bioinformatics analysis identified SERPINA3 and miR-2116-3p.** (A) The expression of SERPINA3 in GBM was analyzed by GEPIA. (B) miR-2116-3p was a common miRNA in starBase and TargetScan. starBase, a tool to predict miRNAs binding to HOXA-AS2. TargetScan, a tool to predict miRNAs binding to SERPINA3.
