# Supplementary material for: HOXA-AS2 enhances GBM cell malignancy by suppressing miR-2116-3p thereby upregulating SERPINA3
Source: BMC Cancer. 2022 Apr 6;22:366. doi: 10.1186/s12885-022-09462-y (PMC8985346; doi:10.1186/s12885-022-09462-y)
Supplement: Supplementary file 2 — Additional file 2. Explaination. [file 12885_2022_9462_MOESM2_ESM.docx]

In this study, we used SERPINA3 (Cat#: ab184567, Abcam, UK), anti-Bax (1:1000, Cat#: ab53154, Abcam, UK), anti-Bcl-2 (1:1000, Cat#: ab32124, Abcam, UK), anti-caspase-3 (1:1000, Cat#: ab32351, Abcam, UK), anti-cleaved caspase-3 (1:1000, Cat#: ab2302, Abcam, UK) and GAPDH (Cat#: ab8245, Abcam, UK) during western blotting assay. Therefore, the original images of blots have been cut depending on the molecular sizes of different antibodies. Besides, the protein marker to prove the molecular sizes of antibodies cannot be vitalized in some original images so that we photographed the bright field to visualize the protein marker.
