# Supplementary material for: HOXA-AS2 enhances GBM cell malignancy by suppressing miR-2116-3p thereby upregulating SERPINA3
Source: BMC Cancer. 2022 Apr 6;22:366. doi: 10.1186/s12885-022-09462-y (PMC8985346; doi:10.1186/s12885-022-09462-y)

Figure 2C-A172 bax

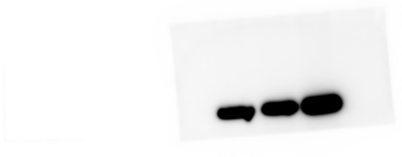

Figure 2C-A172 bax-bright field

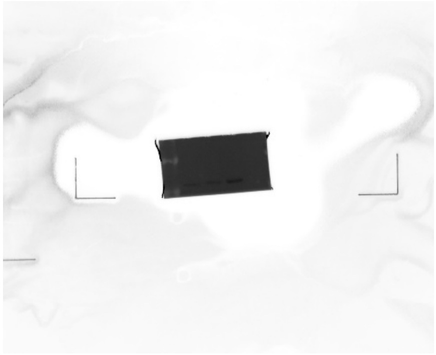

Figure 2C-A172 bcl-2

Figure 2C-A172 bcl-2-bright field

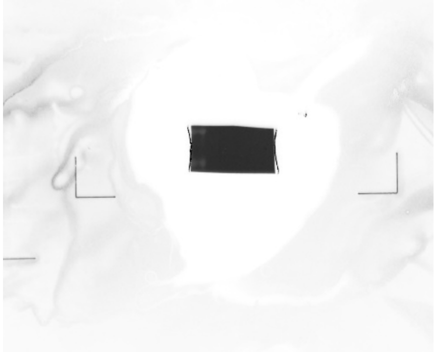

Figure 2C-A172 caspase3

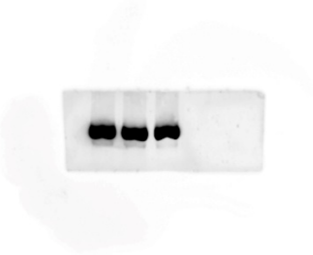

Figure 2C-A172 caspase3-bright field

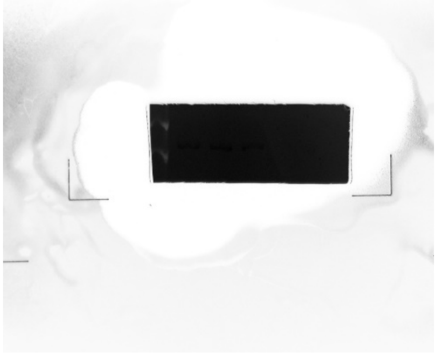

Figure 2C-A172 c-caspase3

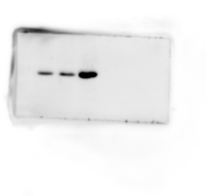

Figure 2C-A172 c-caspase3-bright field

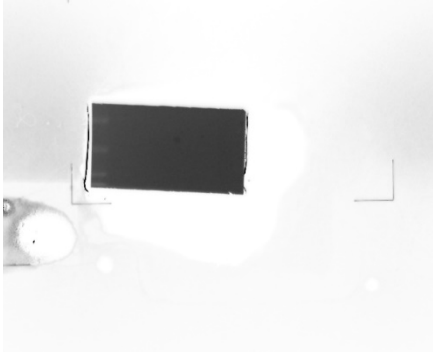

Figure 2C-A172 GAPDH

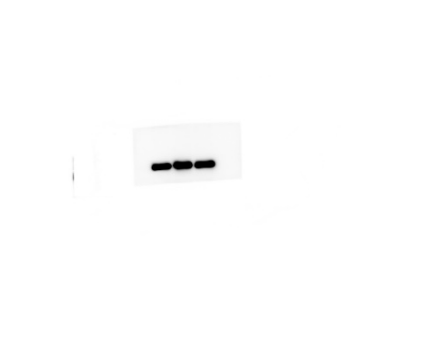

Figure 2C-A172 GAPDH-bright field

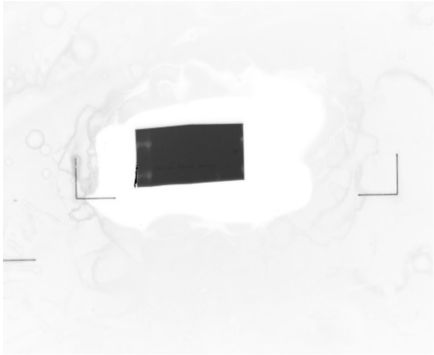

Figure 2C-A172 GAPDH-bright field

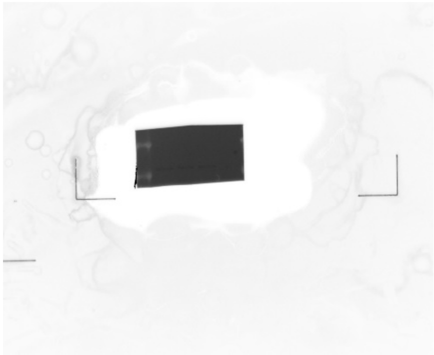

Figure 2C-U251 bax

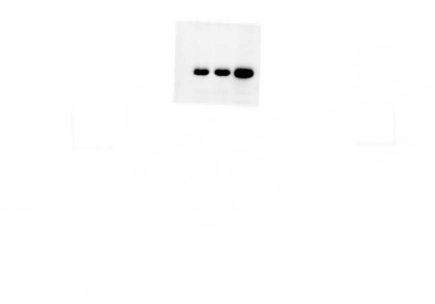

Figure 2C-U251 bax-bright field

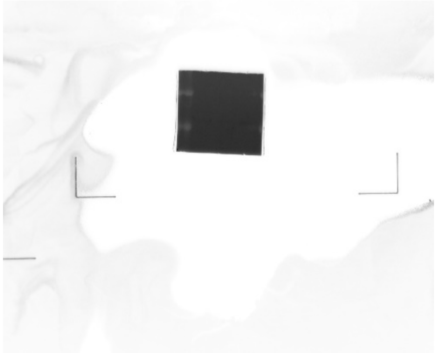

Figure 2C-U251 bcl-2

Figure 2C-U251 bcl-2-bright field

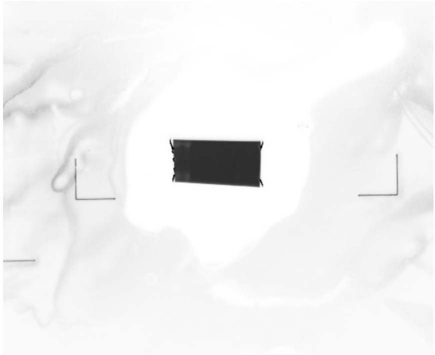

Figure 2C-U251 caspase3

Figure 2C-U251 caspase3-bright field

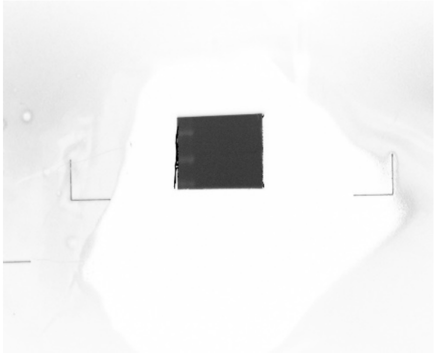

Figure 2C-U251 c-caspase3

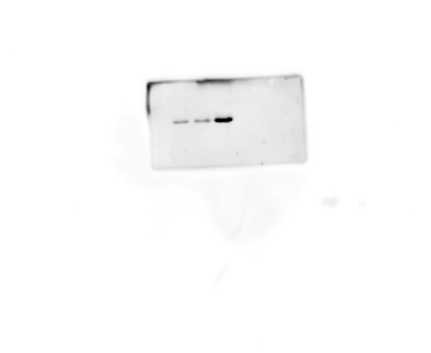

Figure 2C-U251 c-caspase3-bright field

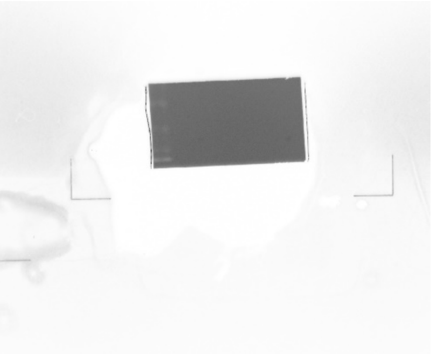

Figure 2C-U251 GAPDH

Figure 2C-U251 GAPDH-bright field

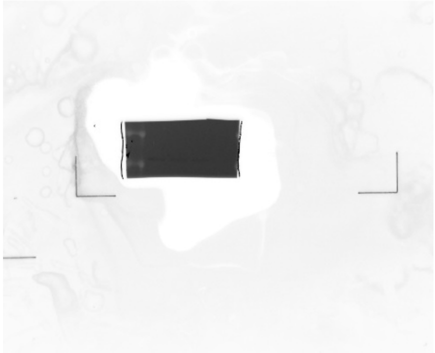

Figure 5-A172 bax

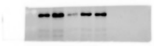

Figure 5-A172 bax-bright field

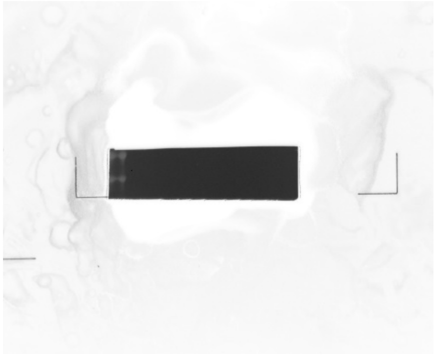

Figure 5-A172 bcl-2

Figure 5-A172 bcl-2-bright field

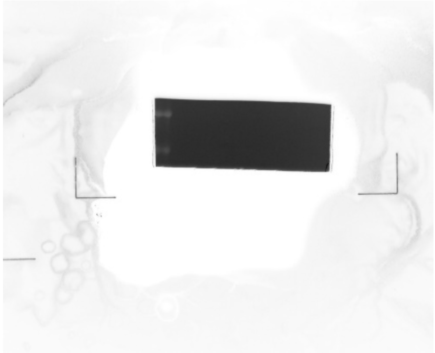

Figure 5-A172 GAPDH

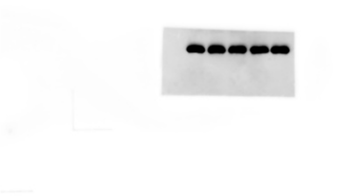

Figure 5-A172 GAPDH-bright field

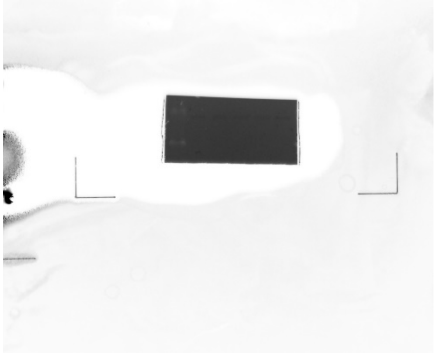

Figure 5C-A172 caspae3

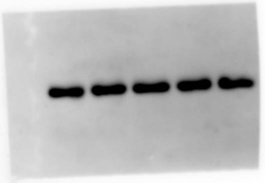

Figure 5C-A172 caspae3-bright field

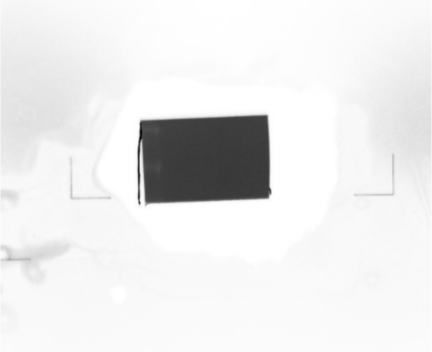

Figure 5C-A172 c-caspase3

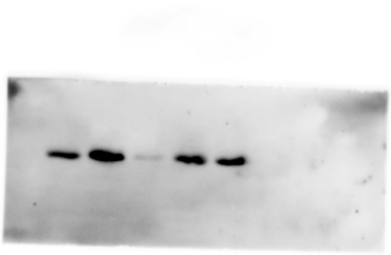

Figure 5C-A172 c-caspase3-bright field

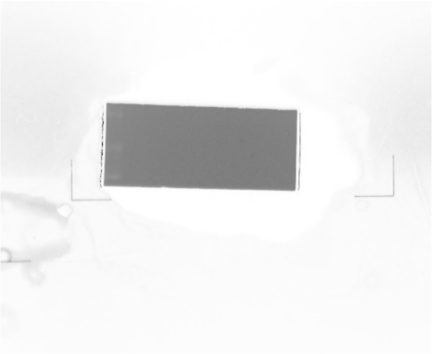

Figure 5C-U251 caspase3

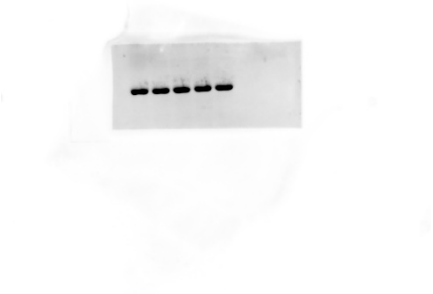

Figure 5C-U251 caspase3-bright field

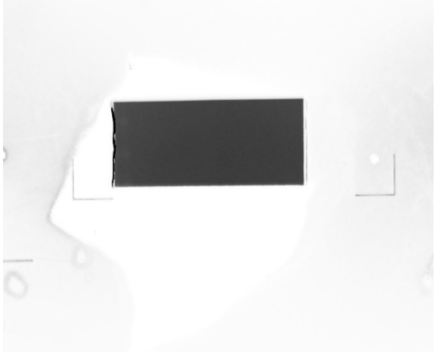

Figure 5C-U251 c-caspase3

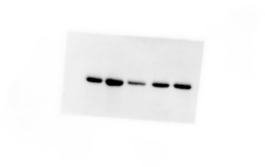

Figure 5C-U251 c-caspase3-bright field

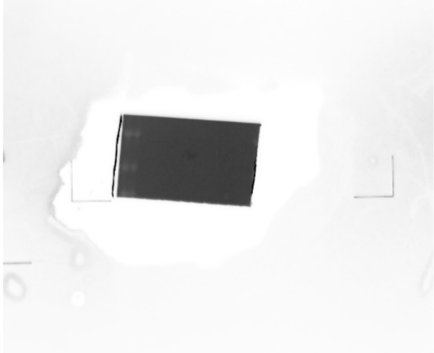

Figure 5-U251 bax

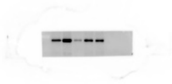

Figure 5-U251 bax-bright field

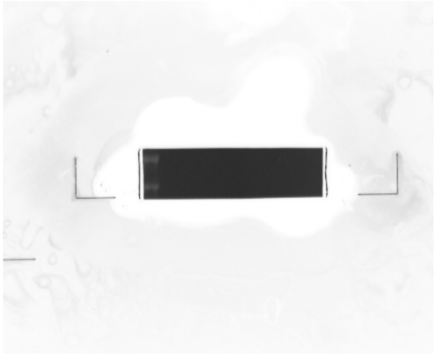

Figure 5-U251 bcl-2

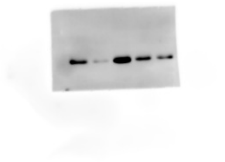

Figure 5-U251 bcl-2-bright field

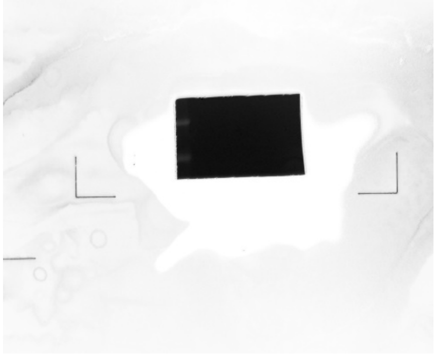

Figure 5-U251 GAPDH

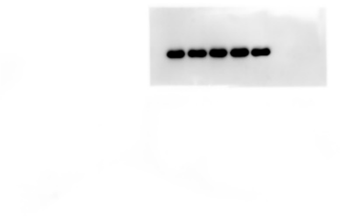

Figure 5-U251 GAPDH-bright field

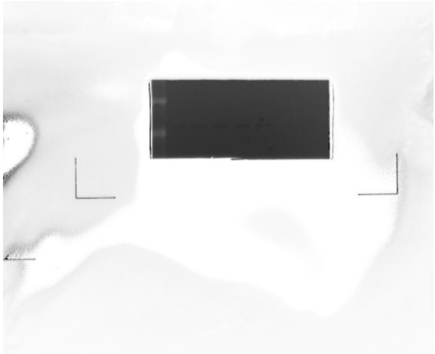

Figure 7G-A172 GAPDH

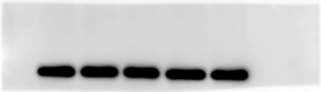

Figure 7G-A172 GAPDH-bright field

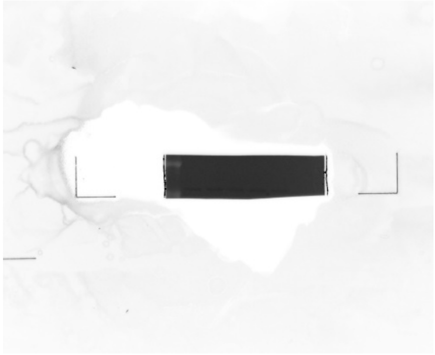

Figure 7G-A172 SERPINA3

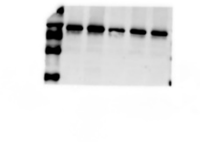

Figure 7G-GAPDH

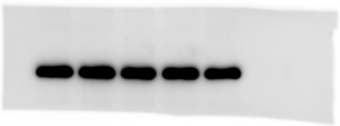

Figure 7G-GAPDH-bright field

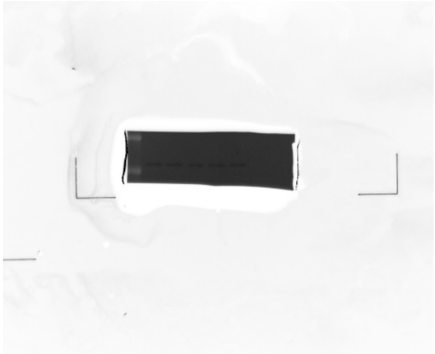

Figure 7G-U251 SERPINA3

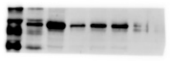

Figure 8C-A172 caspase3

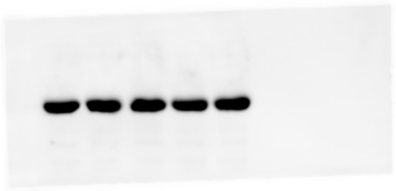

Figure 8C-A172 caspase3-bright field

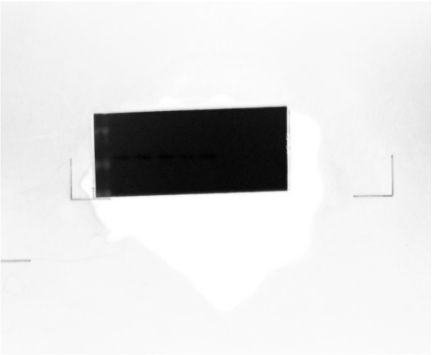

Figure 8C-A172 c-caspase3

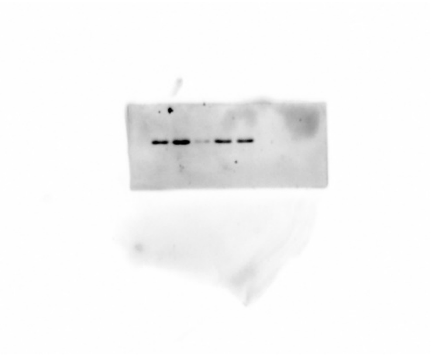

Figure 8C-A172 c-caspase3-bright field

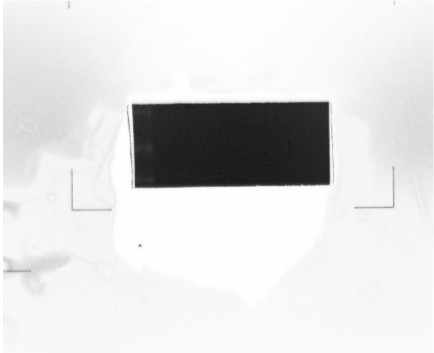

Figure 8C-U251 caspase3

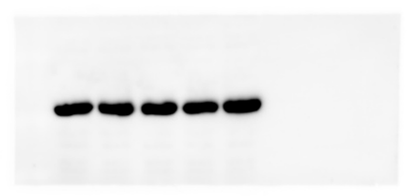

Figure 8C-U251 caspase3-bright field

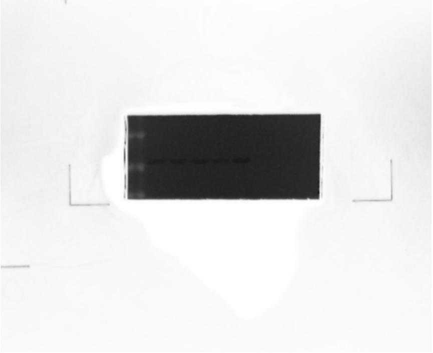

Figure 8C-U251 c-caspase3

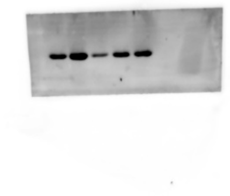

Figure 8C-U251 c-caspase3-bright field

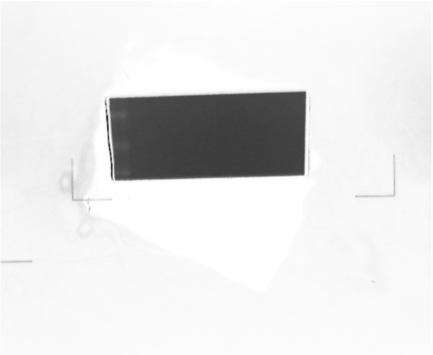

Figure8C-A172 bax

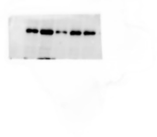

Figure8C-A172 bax-bright field

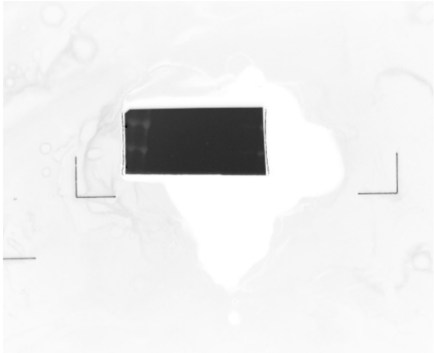

Figure8C-A172 bcl-2

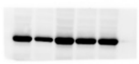

Figure8C-A172 bcl-2-bright field

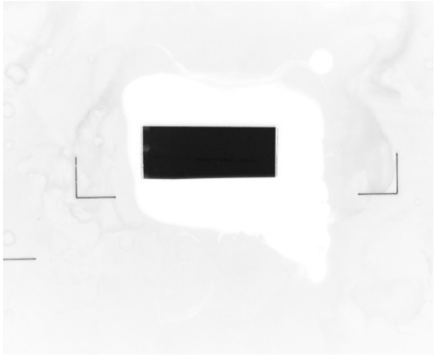

Figure8C-A172 GAPDH

Figure8C-A172 GAPDH-bright field

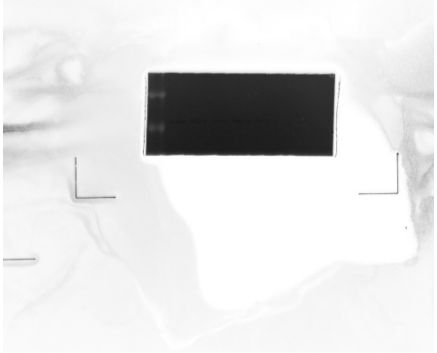

Figure8C-U251 bax

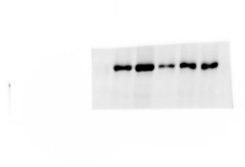

Figure8C-U251 bax-bright field

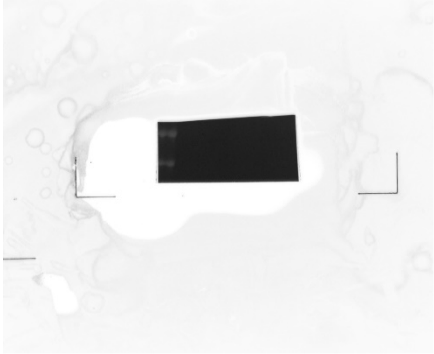

Figure8C-U251 bcl-2

Figure8C-U251 bcl-2-bright field

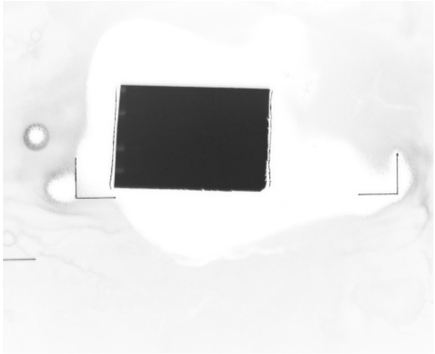

Figure8C-U251 GAPDH

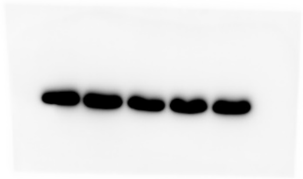

Figure8C-U251 GAPDH-bright field

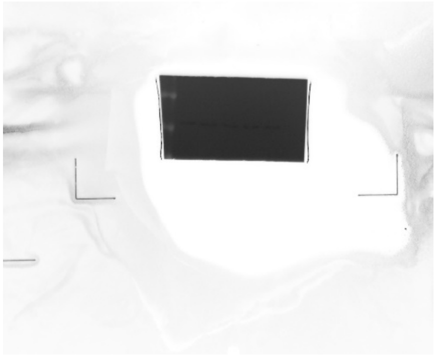

Supplement: Supplementary file 3 — Additional file 3. Western blotting. [file 12885_2022_9462_MOESM3_ESM.pdf]
